# Liberty and Tuberculosis

**Published:** 1898-12-31

**Authors:** 


### Liberty and Tuberculosis.

No great object can be accomplished without great efforts, and no object which for its accomplishment demands the co-operation of large numbers of people, entails much trouble, and even involves restrictions of accustomed liberties, has any chance of success unless the whole mass of the people are taught its necessity and are moved to a certain enthusiasm in its favour. Hence the importance of the remarks made by Sir William Broadbent at Marlborough House when, speaking of the efforts now being made to lessen the prevalence of tuberculosis, he said, "the public attention must be captured, the public imagination must be impressed, the defensive instincts of the general public must be aroused." It is, indeed, only thus that an effective crusade against consumption can be undertaken, for if it is to be successful it must involve drastic measures, and, perhaps, more interference with personal liberty than some people imagine. Man, naturally a nomad, has become a house dweller, and has in course of ages surrounded himself with animals which in varying degrees he has turned into house dwellers also, and all these have had to pay the same penalty, and have become affected with the same disease—tuberculosis. It is then in the house, the home, and the workshop that the difficulty lies.

We ticket the disease with a certain name and treat it as a separate malady; but, in truth, the bacillus which causes it takes its place in Nature along with other moulds and fungi, things the germs of which are so widely spread that they are pretty sure to come sooner or later if only the conditions for their growth are present, and which certainly will not flourish if the conditions are adverse to their growth. The matter is sufficiently simple. Put a pot of jam in a cupboard that is damp and warm and it will go mouldy in a fortnight. Put men to live together in close, insanitary dwellings, ensure that the access of air is deficient, that they shall breathe it over and over again, take care that the place is not thoroughly cleaned, and that there is sufficient warmth and dampness to enable the bacillus to keep alive while sticking to walls and clothes and household utensils, and thus be spread from patient to patient, and your men will grow as mouldy as your jam pots, only we call the mould by another word, and speak of it as consumption. While, then, we go fully with those who would urge the necessity for putting every possible limitation to every known mode of infection, we see a far wider field for preventive measures than those indicated by the programme of the National Association for the Prevention of Consumption. The objects of this association are described by Sir William Broadbent as being (1) to educate the public as to the means of preventing the spread of consumption from those already suffering from the disease; (2) to extinguish tuberculosis in cattle; (3) to promote the erection of sanatoria for the open-air treatment of tuberculous disease. All of them most worthy objects. Most excellent, but not enough. Let us go back to our jam pots and see how next to impossible it is to keep mould at bay so long as the condition of the cupboard is unchanged. Let us consider other diseases caused by other bacilli of whose life-history and variations we perhaps know more than we do about the bacillus of tuberculosis, and note how the virulence of some may fade, and yet be roused again to full activity in appropriate surroundings, and when we recall these things let us not be too sure that consumption will be stayed by measures which solely strive to lessen the chances of the infection being transmitted.

It is the conditions conducive to consumption that we have to combat, not the infection only; and thus we are brought face to face with an enormous question, namely, that of altering the conditions of life of man and the domestic animals. The policy of slaughter is now being much discussed, and there are those who would go needle in hand testing all our herds with tuberculin, and slaughtering those found to be infected. That is the extremest view; but what would even such measures do while the cows are kept in unhealthy byres—damp, ill-drained, devoid of light and ventilation—in which they remain imprisoned without exercise so long as milk will flow? These are the conditions which lead to tuberculosis, and killing a few thousand cattle does nothing to alter them. And so with man; removal of the infected, care as to expectoration, the boiling of milk, and all the rest of it, will do much. They are all useful measures, but they do not alter the conditions of life, and to alter the conditions of life is—well, is almost a social revolution, and one involving the loss of much that goes by the name of "liberty."

